# Blockade of platelet-derived growth factor receptor-β, not receptor-α ameliorates bleomycin-induced pulmonary fibrosis in mice

**DOI:** 10.1371/journal.pone.0209786

**Published:** 2018-12-31

**Authors:** Masami Kishi, Yoshinori Aono, Seidai Sato, Kazuya Koyama, Momoyo Azuma, Shuichi Abe, Hiroshi Kawano, Jun Kishi, Yuko Toyoda, Hiroyasu Okazaki, Hirohisa Ogawa, Hisanori Uehara, Yasuhiko Nishioka

**Affiliations:** 1 Department of Respiratory Medicine and Rheumatology, Graduate School of Biomedical Sciences, Tokushima University, Tokushima, Japan; 2 Department of Pathology and Laboratory Medicine, Graduate School of Biomedical Sciences, Tokushima University, Tokushima, Japan; Helmholtz-Zentrum Munich, GERMANY

## Abstract

Platelet-derived growth factor (PDGF) has been implicated in the pathogenesis of pulmonary fibrosis. Nintedanib, a multi-kinase inhibitor that targets several tyrosine kinases, including PDGF receptor (PDGFR), was recently approved as an anti-fibrotic agent to reduce the deterioration of FVC in patients with idiopathic pulmonary fibrosis (IPF). However, the effects of PDGFR-α or -β on pulmonary fibrosis remain unclear. In an attempt to clarify their effects, we herein used blocking antibodies specific for PDGFR-α (APA5) and -β (APB5) in a bleomycin (BLM)-induced pulmonary fibrosis mouse model. The effects of these treatments on the growth of lung fibroblasts were examined using the ^3^H-thymidine incorporation assay *in vitro*. The anti-fibrotic effects of these antibodies were investigated with the Ashcroft score and collagen content of lungs treated with BLM. Their effects on inflammatory cells in the lungs were also analyzed using bronchoalveolar lavage fluid. We investigated damage to epithelial cells and the proliferation of fibroblasts in the lungs. APA5 and APB5 inhibited the phosphorylation of PDGFR-α and -β as well as the proliferation of lung fibroblasts induced by PDGF-AA and BB. The administration of APB5, but not APA5 effectively inhibited BLM-induced pulmonary fibrosis in mice. Apoptosis and the proliferation of epithelial cells and fibroblasts were significantly decreased by the treatment with APB5, but not by APA5. The late treatment with APB5 also ameliorated fibrosis in lungs treated with BLM. These results suggest that PDGFR-α and -β exert different effects on BLM-induced pulmonary fibrosis in mice. A specific approach using the blocking antibody for PDGFR-β may be useful for the treatment of pulmonary fibrosis.

## Introduction

Idiopathic pulmonary fibrosis (IPF) is a progressive and lethal fibrotic disease of the lungs [1]. The pathogenesis of IPF involves the proliferation of mesenchymal cells and collagen deposition after the deregulation of repair from epithelial cell damage. Since fibrogenesis is regulated by various growth factors, targeting these molecules has potential as a strategy to treat patients with pulmonary fibrosis [2]. Among these growth factors, platelet-derived growth factors (PDGFs) play a major role in pulmonary fibrosis by stimulating the growth and migration of lung fibroblasts [3,4]. Therefore, the inhibition of PDGF receptors may improve or slow the progression of IPF.

In animal models, we and others have demonstrated that treatments with imatinib, an inhibitor of PDGFR-α and -β, c-KIT, and Bcr-Abl, inhibited the development of pulmonary fibrosis in radiation- and bleomycin (BLM)-induced models [5–7]. These findings suggest that targeting the PDGF/PDGFR axis has potential in the treatment of IPF; however, clinical trials with imatinib for patients with IPF failed to show any anti-fibrotic effects [8]. Another multiple kinase inhibitor, nintedanib, which inhibits several receptor tyrosine kinases for PDGFs, fibroblast growth factors, and vascular endothelial growth factor, was found to reduce the deterioration of forced vital capacity (FVC) and acute exacerbation in patients with IPF [9]. Wollin et al. reported that nintedanib inactivated human lung fibroblasts through the inhibition of PDGFR-α and -β, and the IC50 of the nintedanib to inhibit the autophosphorylation of PDGFR-α and -β was 20-50-fold less than that of imatinib [10]. The successful findings reported for nintedanib highlight the importance of the PDGF/PDGFR axis as a therapeutic target for IPF.

It currently remains unclear whether the selective inhibition of PDGFR is sufficient to reduce pulmonary fibrosis by targeting a single molecule, and also if PDGFR-α or -β is a better target for reducing fibrogenesis in the lungs. Based on phenotypic analyses of gene knockouts, previous studies reported that PDGF isoforms and PDGFRs have different physiological functions during the embryonic period [11]. Bostrӧm et al. reported that PDGF-A knockout and partially rescued PDGFR-α null mutants displayed a lung emphysema-like phenotype due to failed alveolar septum formation, indicating that the blockade of PDGFR-α leads to a loss of integrity in the lungs [12]. In addition, targeted therapy with a specific antibody may reduce the adverse events caused by multi-targeting compounds including imatinib and nintedanib [8,9].

We herein examined the effects of PDGFR-α and -β using specific blocking antibodies for mouse PDGFR-α and -β in a BLM-induced pulmonary fibrosis mouse model.

## Materials and methods

### Animals and materials

Five-week-old female C57BL/6 mice were purchased from Charles River Japan, Inc. (Yokohama, Japan). They were maintained in the animal facility of Tokushima University under specific pathogen-free conditions according to the guidelines of the ethics committee of our university [6]. The present study was approved by Institutional Animal Care and Use Committee of the Tokushima University (Permission Number: 08128). The anti-mouse PDGFR blocking antibodies APA5 and APB5 were kindly provided by Dr. Nishikawa (Kyoto University). MLE12 cells were purchased from the American Type Culture Collection (Manassas, VA). Bovine serum albumin, IgG from rat serum, and PDGF-AA and PDGF-BB were purchased from Sigma-Aldrich (St. Louis, MO). BLM was purchased from Nippon Kayaku Co. (Tokyo, Japan).

### Fibroblast isolation

Mouse lung fibroblasts were obtained according to a previously reported method [13]. The lungs were harvested from untreated mice and minced with scissors. Minced lungs were digested with 0.5% trypsin. Harvested cells were cultured in RPMI1640 medium supplemented with 10% fetal bovine serum (FBS) (GIBCO BRL, Rockville, MD) for several days. Proliferating cells (doubling time in 10% FBS: 15 to 18 hours) were used as lung fibroblasts at five to 10 passages. These fibroblasts were positively stained with anti-vimentin and α-smooth muscle actin antibodies, which indicate a myofibroblast phenotype, and negatively stained with an anti-cytokeratin antibody, which indicates an epithelial cell phenotype.

### Epithelial cell isolation

Lungs harvested from mice treated with or without BLM were minced with scissors [14]. Minced lungs were digested with DNase 1 (Chemicon (Merck Millipore), Darmstadt, Germany), collagenase A (Roche, Basel, Switzerland), and Dispase 2 (Roche, Basel, Switzerland). Red blood cells were removed from single cell suspensions using ACK RBC lysis buffer (G-Biosciences, Saint Louis, MO). Thereafter, we used MACS (Miltenyi Biotec, Bergisch Gladbach, Germany) to isolate type 2 epithelial cells, according to the manufacturer’s instructions. After the negative selection of leukocytes with CD45 microbeads (Miltenyi Biotec), type 2 epithelial cells were positively selected using a FITC anti-mouse CD326 (Ep-CAM) antibody (Biolegend, San Diego, CA) and anti-FITC microbeads (Miltenyi Biotec). The purity of isolated cells was assessed by immunocytochemistry. Cells were incubated with a 1:800 dilution of a PE/Cy7 rat anti-mouse CD326 (Ep-CAM) antibody as the primary antibody, and an Alexa Fluor 488 chicken anti-rat IgG antibody (Invitrogen, Eugene, OR) was used as the secondary antibody. Nuclei were counterstained with 4’-6-diamidino-2-phenylindole (DAPI). The ratio of Ep-CAM-positive cells was more than 60% of the selected cells.

### Immunoblotting

Immunoblotting was performed for the detection of PDGFR-α and -β and phosphorylated PDGFR-α and -β using cell lysates of mouse lung fibroblasts as described previously [6]. Cells were cultured in 6-well plates in RPMI1640 supplemented with 0.1% FBS. After APA5 (1 μg/ml) or APB5 (1 μg/ml) was administered, they were incubated for 1 hour and stimulated with PDGF-AA (10 ng/ml) or PDGF-BB (10 ng/ml) for 10 minutes, respectively. Cells were lysed with 1 ml of lysis buffer [1%NP40, 25 mM TrisHCl (pH7.4), 10 mM sodium pyrophosphate, 100 mM sodium fluoride, 10 mM EDTA, 10 mM EGTA, and 1 mM phenylmethylsulfonyl fluoride (PMSF)]. Protein concentrations were measured by a protein assay (Bio-Rad Laboratories, Hercules, CA). These samples were boiled for 3 minutes and run on 7.5% SDS-PAGE. Proteins were then blotted on Clear Blot membrane-P (Atto. Corp., Tokyo, Japan). The membrane was blocked with blocking one (Nacalai Tesque, Inc., Kyoto, Japan) at RT for 1 hour and incubated at 4°C overnight with primary antibodies. The membrane was then incubated with secondary antibodies at RT for 1 hour. The blot was developed using the enhanced chemiluminescence (ECL) method (GE Healthcare, Ltd., Amersham, UK).

The first antibodies used were as follows: anti-phospho-PDGFR alpha (Tyr1018) antibody (1:1000 dilution, #4547; Cell Signaling Technology, Inc., MA), anti-PDGFR alpha (phospho Y754) antibody (1:1000 dilution, ab5460; Abcam, Cambridge, UK), phospho-Akt (Ser473) antibody (1:1000 dilution, #9271; Cell Signaling Technology, Inc., MA), PDGF Receptor α Antibody (1:1000 dilution, #3164; Cell Signaling Technology, Inc., MA), PDGF Receptor β (28E1) Rabbit mAb (1:1000 dilution, #3169; Cell Signaling Technology, Inc., MA), AKT antibody (1:1000 dilution, #9272; Cell Signaling Technology, Inc., MA), and anti-β-actin antibody (C4) (1:1000 dilution, sc-47778; Santa Cruz Biotechnology). The second antibodies used were as follows: goat anti-rabbit IgG-HRP (1:5000 dilution, sc-2004; Santa Cruz Biotechnology) and anti-mouse IgG-HRP (1:1000 dilution, GE Healthcare). Densitometric quantification was done by ImageQuant LAS 4000 (GE Healthcare, Fairfield, CT).

### Proliferation assay

Lung fibroblasts were added to a 96-well plate at 8×10^3^ cells per well. Cells (50–60% confluent) were cultured in RPMI1640 medium supplemented with 10% FBS overnight, and then rendered quiescent in RPMI1640 medium containing 0.3% FBS overnight. Cells (60–70% confluent) were then treated with PDGF-AA and BB (10 ng/ml) and various concentrations of APA5 and APB5 (0.01–1 ng/ml) for 72 hours and labeled with ^3^H-TdR (6.7 Ci/mmol; Amersham Corp., Arlington Heights, IL) at 1 μCi/well for the final 18 hours. Cells were harvested on glass filters in the cell harvester, MASHII (Labo Science Co., Tokyo, Japan), and the incorporation of ^3^H-TdR was measured with a liquid scintillation counter.

### Flow cytometry

Flow cytometry was performed for the detection of PDGFR-α and -β in mouse lung fibroblasts and mouse alveolar epithelial cells. Mouse lung fibroblasts were plated onto 6-well plates (1×10^5^/well) and incubated in RPMI1640 medium supplemented with 10% FBS at 37°C under 5% CO2 in humidified air. Cells (5×10^5^) were collected and incubated for 30 minutes on ice with PE anti-mouse CD140a (PDGFR-α) antibody, PE anti-mouse CD140b (PDGFR-β) antibody and purified RAT IgG (Invitrogen). Mouse alveolar epithelial cells were isolated using MACS as described above. Cells were incubated with a FITC anti-mouse CD326 (EpCAM) antibody, PE anti-mouse CD140a (PDGFR-α) antibody, and PE anti-mouse CD140b (PDGFR-β) antibody (Biolegend). Stained cells were analyzed by flow cytometry using BD LSRFortessa for acquisition and BD FACSDiva software 6.0 (BD Biosciences, San Jose, CA) for analyses.

### BLM treatment

Mice were anesthetized with pentobarbiturate, and mini-osmotic pumps (ALZET 2001, DURECT Corporation, CA) containing 200 μl saline with or without BLM (140 mg/kg) were implanted subcutaneously into the left suprascapular lesion through an incision at the base of the neck [15]. BLM was constantly infused with minipumps from day 0 to 7 for 7 days, as described in the manufacturer’s protocol. Each experiment was performed on at least three mice per group.

### Administration of APA5, APB5, and IgG

APA5, APB5, and IgG from rat serum (control) were dissolved in PBS. APA5, APB5, and control rat IgG (1 mg/body) were injected intraperitoneally into each mouse every other day from day 0 to 26. The dose and frequency of injections were selected based on previous studies using APA5 and APB5 in mice [16,17].

### Bronchoalveolar lavage

Regarding bronchoalveolar lavage (BAL), mice were anesthetized with pentobarbital and the lungs and heart were surgically exposed. The trachea was cannulated and the lungs were lavaged 5 times with 0.7 ml of 0.1 mM PBS. BAL cells were collected by centrifugation, and live cells were counted using a hemocytometer. After counting the cell number in BAL fluid, cells were cytospun onto glass slides and stained with Diff-Quik (Sysmex Co., Hyogo, Japan) for differential cell counting.

### Collagen assay

The total amount of collagen in the lungs was assessed using the Sircol Collagen Assay kit (Biocolor Ltd., Belfast, Northern Ireland). On Day 28 after implantation of the minipumps containing BLM, the left lungs were harvested and homogenized in 0.5 M acetic acid (50 volumes to wet lung weight) containing approximately 1 mg pepsin/10 mg tissue residue. Each sample was incubated at room temperature for 24 hours with stirring. After centrifugation, 100 μl of each supernatant was assayed. One milliliter of Sircol dye reagent, which binds to collagen, was added to each sample and then mixed for 30 minutes. After centrifugation, the pellets were suspended in 1 ml of the alkali reagent included in the kit and read at 540 nm using a spectrophotometer. Collagen standard solutions were utilized to construct a standard curve.

### Histopathology

The right lungs were fixed in 10% buffered formalin and embedded in paraffin. Sections (thickness of 3 to 4 μm) were stained with hematoxylin and eosin and then examined by light microscopy. A numerical fibrotic scale was used (Ashcroft score) in a quantitative histological analysis [18]. The severity of fibrotic changes in each lung section was assessed as a mean score of severity from observed microscopic fields. Fifteen fields within each lung section were evaluated at a magnification of ×100. Each field was assessed individually for the severity of fibrotic changes and given a score of 0 (normal) to 8 (total fibrosis). The mean score of all evaluated fields was regarded as the fibrotic score. To evaluate fibrotic changes in more detail, Masson’s trichrome staining was also performed.

### Immunohistochemistry

On day 14 of the BLM treatment, harvested lungs were fixed in 10% buffered formalin and embedded in paraffin. Lung sections (thickness of 3 to 4 μm) were deparaffinized and rehydrated. Antigens were retrieved by heating the sections in citrate buffer. Endogenous peroxidase activity was neutralized with 0.3% H_2_O_2_. Staining was performed using the VECTASATIN universal quick kit (Vector Laboratories, Burlingame, CA) following the manufacturer’s instructions.

To assess cell proliferation in the lungs, a rat anti-mouse Ki-67 antigen monoclonal antibody (Dako, Glostrup, Denmark) was used as the primary antibody. Sections were incubated at RT for 1 hour with a 1:100 dilution of the primary antibody. After washing, they were incubated with a biotinylated anti-rat secondary antibody at RT for 1 hour, followed by an incubation with ready-to-use streptavidin/peroxidase complex reagent for 10 minutes. Staining of the sections was developed with a diaminobenzidine substrate kit (Vector Laboratories) and sections were counterstained with Mayer’s hematoxylin (MUTO PURE CHEMICALS CO., LTD., Tokyo, Japan). Ki-67-positive nuclei in the alveolar wall and in the interalveolar spaces of whole sections were counted.

Regarding the detection of PDGFR expression in lung tissue sections, immunohistochemistry was performed as described above. As the primary antibodies, a 1:100 dilution of a rabbit anti-PDGFR-α antibody (Cell Signaling Technology) and a 1:100 dilution of a rabbit anti-PDGFR-β antibody (Cell Signaling Technology) were used.

Regarding the detection of apoptotic cells, sections were stained using the terminal deoxynucleotidyl transferase-mediated dUTP-biotin nick end labeling (TUNEL) method with the DeadEnd Fluorometric TUNEL System (Promega, Madison, WI) following the manufacturer’s instructions. Nuclei were counterstained with 4’-6-diamidino-2-phenylindole (DAPI). Fluorescein-12-dUTP-labeled DNA was visualized directly by fluorescence microscopy. The number of TUNEL-positive cells per field was counted from 20 random fields at ×20.

Regarding the detection of alveolar type 2 epithelial cells, immunohistochemistry was performed without antigen retrieval. As the primary antibody, a 1:2000 dilution of a rabbit anti-human prosurfactant protein C (proSP-C) polyclonal antibody (Chemicon (Merck Millipore)) was used. proSP-C-positive nuclei in 3 random fields were counted at ×100.

Regarding the detection of PDGFRs and pulmonary epithelial cells or mesenchymal cells, Costainings of PDGFRs and EpCAM or α-SMA was performed. As the primary antibodies, a rabbit anti-PDGFR-α antibody (Cell Signaling Technology) and a rabbit anti-PDGFR-β antibody (Cell Signaling Technology) and a FITC anti-mouse CD326(Ep-CAM) antibody (Biolegend) or anti-alpha smooth muscle actin antibody (abcam, Cambridge, UK) were used as the primary antibodies. Sections were incubated overnight at 4°C with a 1:50 dilution of the primary antibodies. After washing, they were incubated with a goat anti-rabbit IgG (H+L) cross-adsorbed secondary antibody, Alexa Fluor 594 (Invitrogen) and donkey anti-goat IgG (H+L) highly cross-adsorbed secondary antibody, Alexa Fluor Plus 488 (Invitrogen) at RT for 1 hour. Nuclei were counterstained with VECTASHIELD Antifade Mounting Medium with DAPI (Vector Laboratories). They were visualized directly by fluorescence microscopy.

### Statistical analysis

Comparisons among multiple groups were performed using a one-way analysis of variance with the Newman-Keuls post hoc correction (GraphPad Prism, version 5.0; GraphPad Software, Inc., San Diego, CA). Differences were significant if p values were less than 0.05.

## Results

### APA5 and APB5 specifically inhibit the proliferation of lung fibroblasts stimulated with PDGF-AA or -BB

**Fig 1:**
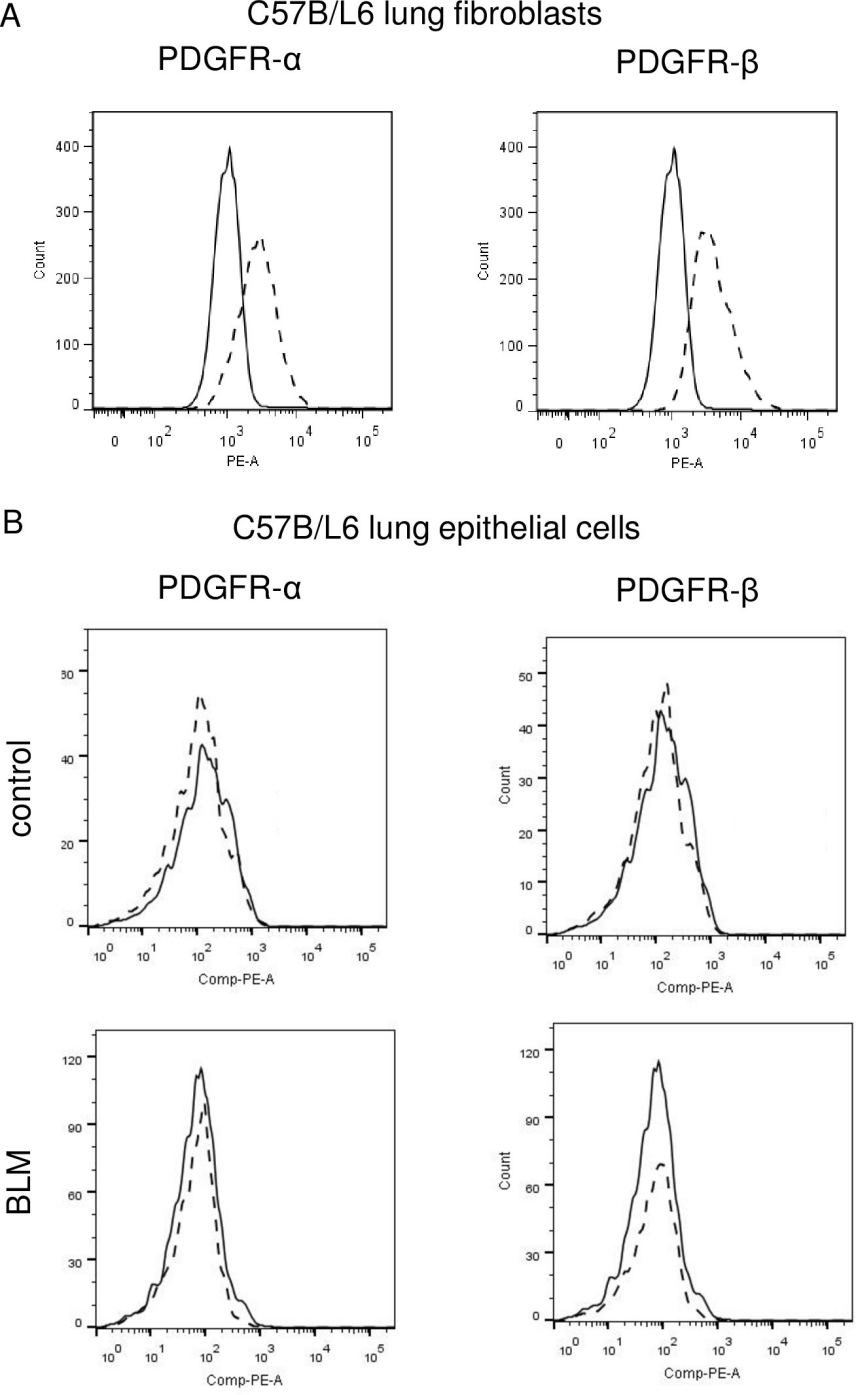
Expression of PDGFR-α and -β by murine lung fibroblasts and lung epithelial cells. (A) A flow cytometric analysis showed that PDGFR-α and -β were both detected on C57B/L6 lung fibroblasts. (B) Mouse alveolar type 2 epithelial cells were isolated as described in the Materials and Methods section. The cells obtained were investigated by a FACS analysis. Neither PDGFR-α nor -β was detected in freshly isolated mouse alveolar type 2 epithelial cells. Similar results were obtained at least two independent experiments.

We examined the expression of PDGFR-α and -β in murine lung fibroblasts using a flow cytometric analysis. As shown in Fig 1A and S1A Fig, lung fibroblasts derived from C57BL/6 mice expressed PDGFR-α and -β (positive rate; 64.5% and 81.6%, respectively). The expressions of PDGFR-α and -β were also confirmed in another lung fibroblast cell line CCL206 (S1C Fig). On the other hand, when we examined the alveolar epithelial cells purified from mice as EpCAM-positive cells, the expressions of PDGFRs were not detected by flow cytometric analysis even if cells were purified after bleomycin treatment (Fig 1B, S1B Fig).

**Fig 2:**
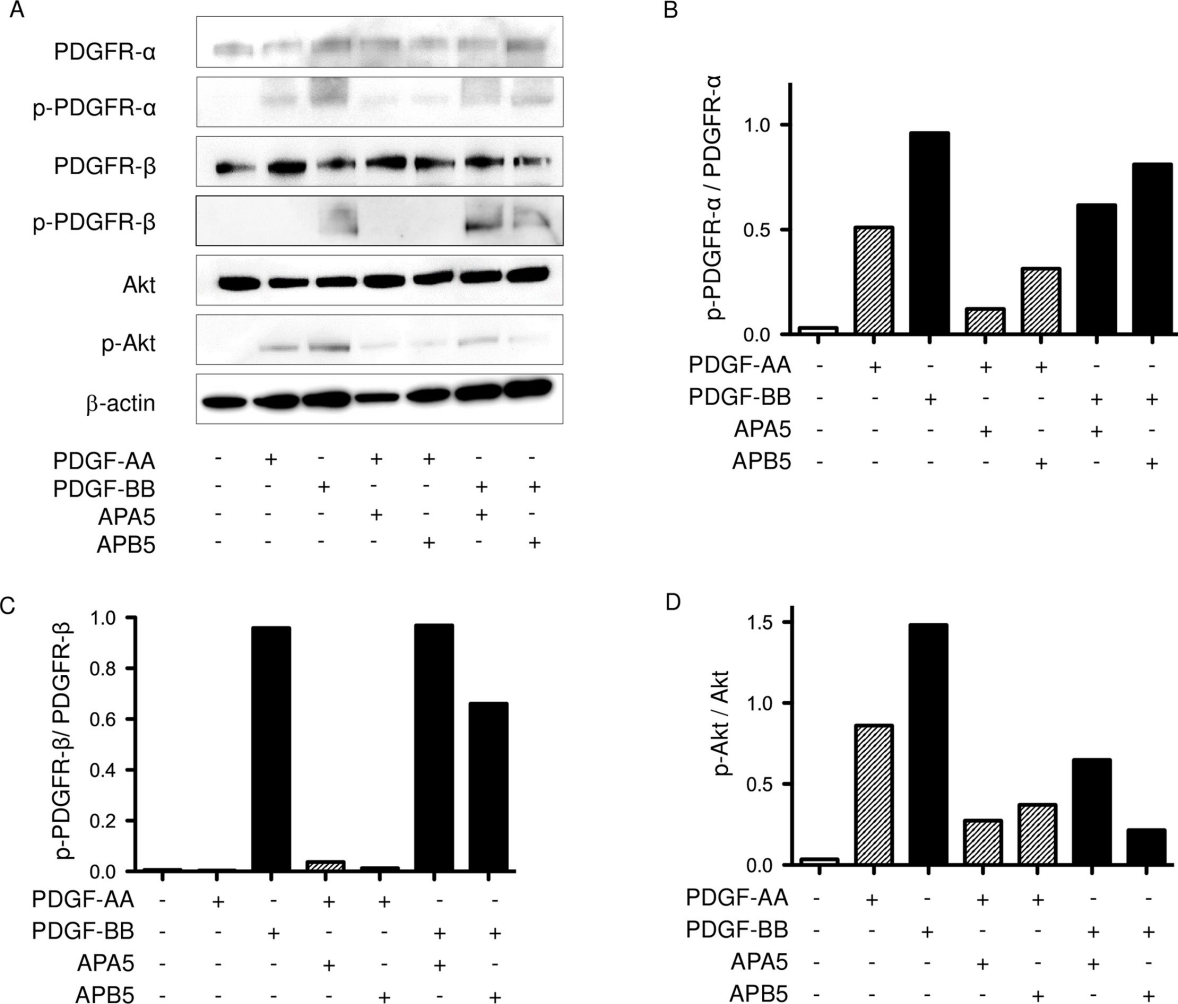
The inhibitory effects of APA5 and APB5 on the phosphorylation of PDGFR-α -β, and Akt. Immunoblotting was performed for the detection of PDGFRs, Akt, and their phosphorylation in lung fibroblasts. Murine lung fibroblasts were stimulated with PDGF-AA (10 ng/ml) and -BB (10 ng/ml) in the presence of the anti-PDGF-α antibody APA5, anti-PDGFR-β antibody APB5, or control Ab. APA5 inhibited the phosphorylation of PDGFR-α induced by PDGF-AA. APB5 inhibited the phosphorylation of PDGFR-β induced by PDGF-BB. The phosphorylation of Akt was also inhibited by both antibodies. Representative immunoblot (A) and corresponding densitometric quantification of PDGFRs phosphorylation and Akt phosphorylation (B-D) are shown.

Next, we examined the inhibitory effects of APA5 and APB5 on the signaling pathway of PDGFR-α and -β (Fig 2, S2 Fig). Addition of PDGF-AA induced the phosphorylation of PDGFR-α, but not PDGFR-β. On the other hand, PDGF-BB phosphorylated both PDGFR-α and -β. APA5 inhibited the phosphorylation of PDGFR-α induced by both PDGF-AA and PDGF-BB and did not blocked the phosphorylation of PDGFR-β induced by PDGF-BB. APB5 blocked the phosphorylation of PDGFR-β induced by PDGF-BB. In addition, APB5 reduced the phosphorylation of PDGFR-α induced by PDGF-AA, although the level of inhibition was weaker than APA5. The total inhibitory activity of PDGFR-α and -β by APA5 and APB5 was confirmed by the phosphorylation of Akt. These results demonstrated that APA5 inhibited the signaling of PDGFR-α, but not PDGFR-β. APB5 blocked the signaling of PDGFR-β, although the signaling pathway of PDGFR-α was also affected by APB5.

**Fig 3:**
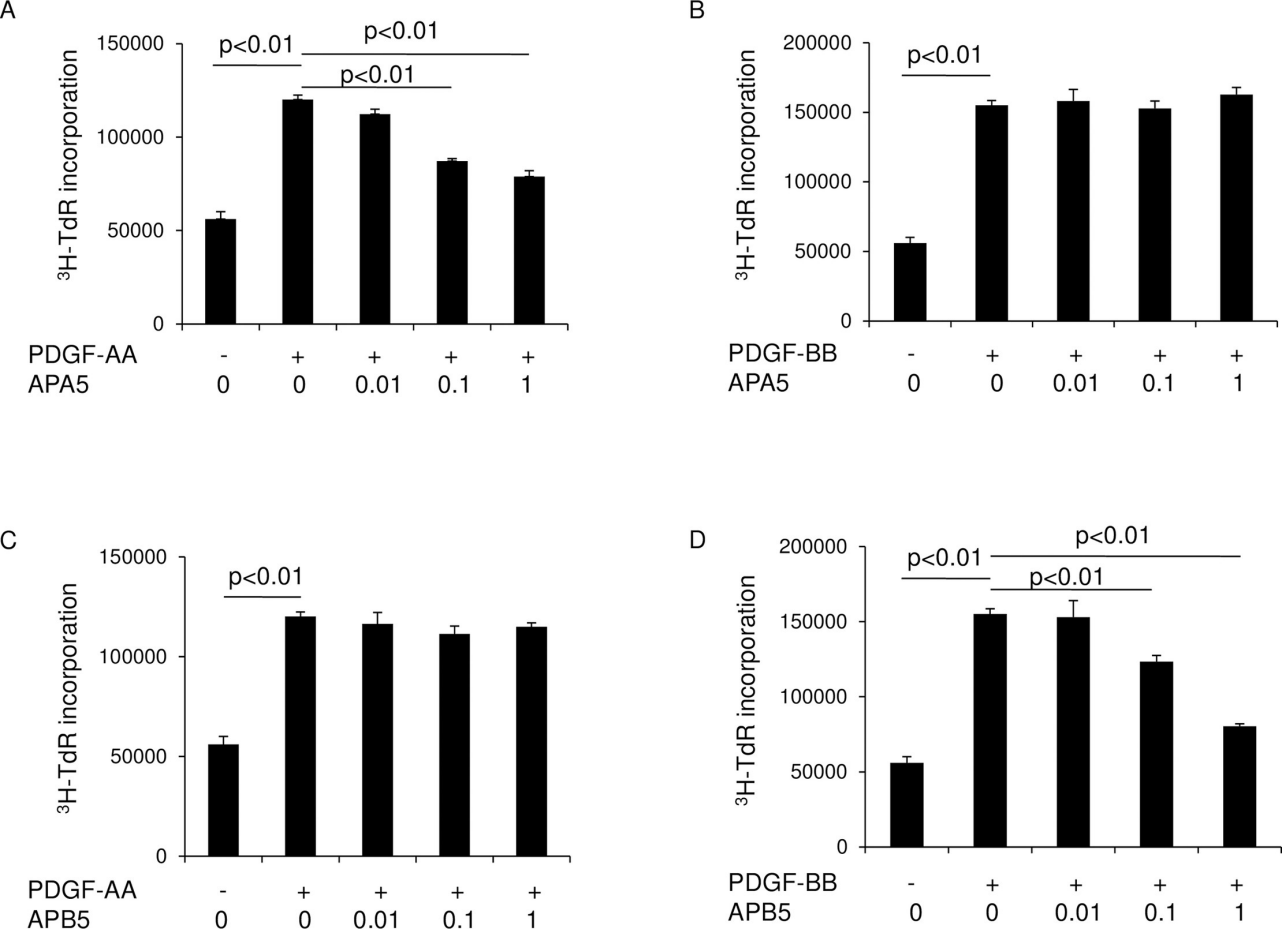
Specific inhibitory effects of APA5 and APB5 on the proliferation of lung fibroblasts. Lung fibroblasts were treated with PDGF-AA (10 ng/ml) and -BB (10 ng/ml) in the presence or absence of various concentrations of APA5 and APB5 (0.01–1 μg/ml), and then labeled with ^3^H-TdR at 1 μCi/well. The incorporation of ^3^H-TdR was measured with a liquid scintillation counter. PDGF-AA and -BB induced the proliferation of murine lung fibroblasts. APA5 inhibited the proliferation of fibroblasts induced by PDGF-AA in a dose-dependent manner (A), but did not inhibit that induced by PDGF-BB (B). APB5 inhibited the proliferation of fibroblasts induced by PDGF-BB in a dose-dependent manner (D), but did not inhibit that induced by PDGF-AA (C).

We then examined the inhibitory effects of APA5 and APB5 on the proliferation of lung fibroblasts using the ^3^H-thymidine incorporation assay (Fig 3). The treatment with APA5 inhibited the proliferation of fibroblasts induced by PDGF-AA in a dose-dependent manner, but not that induced by PDGF-BB. APB5 showed the inhibition of the growth of lung fibroblasts induced by PDGF-BB, but not by PDGF-AA. These results indicated the biological specificity of APA5 or APB5 in blocking PDGFR.

### Treatment with APB5 attenuates pulmonary fibrosis in BLM-treated mice

**Fig 4:**
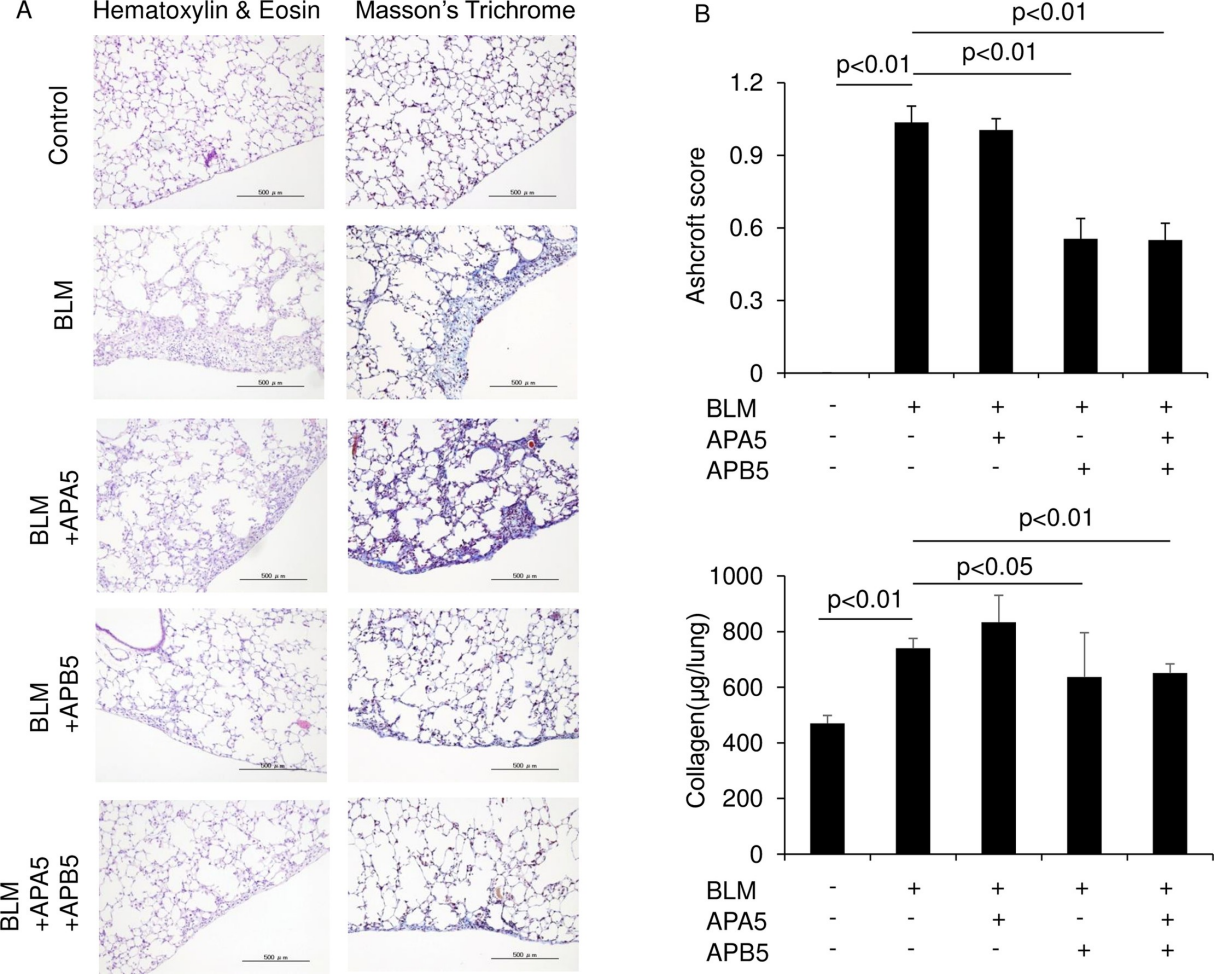
Treatment with APB5, but not APA5 attenuates pulmonary fibrosis in BLM-treated mice. BLM was injected subcutaneously with a mini-osmotic pump for 7 days. APA5, APB5, and IgG as a control were injected intraperitoneally every other day from the start on Day 0 to sacrifice on Day 28. (A) Representative photomicrographs of hematoxylin & eosin and Masson’s trichrome staining of the lungs on Day 28. BLM-treated mice showed fibrosis and collagen deposition in the subpleural areas of the lungs. The administration of APB5 attenuated fibrosis from that in BLM-treated mice. However, the administration of APA5 did not exert any anti-fibrotic effects. Magnification ×100. (B) In the quantitative histological analysis, the severity of fibrotic changes in each lung section was assessed by the Ashcroft score and the collagen assay on Day 28. The administration of APB5 significantly decreased the Ashcroft score and the collagen content from that in BLM-treated mice. Data are presented as means ± SEM. N = 4 for the control and N = 4–7 for the BLM treatment groups.

We investigated the anti-fibrotic effects of APA5 and APB5 in the BLM-induced pulmonary fibrosis mouse model. APA5, APB5, and control Ab were injected intraperitoneally every other day from Day 0 to sacrifice on Day 28. Fibrogenesis in the lungs was evaluated by Hematoxylin & Eosin (Ashcroft score) and Masson’s Trichrome staining of the lungs. A histological analysis of BLM-treated mice revealed fibrosis and collagen deposition in the subpleural areas of the lungs. The administration of APB5 significantly decreased fibrotic changes from those with control Ab (Fig 4A and 4B). The anti-fibrotic effects of APB5 was also confirmed by the quantification of collagen content in the lungs (Fig 4B). However, APA5 did not attenuate pulmonary fibrosis induced by BLM. In addition, the treatment with both APA5 and APB5 did not enhance anti-fibrotic effects over those with APB5 alone.

### APB5, but not APA5, inhibited the proliferation of lung fibroblasts in lungs treated with BLM

**Fig 5:**
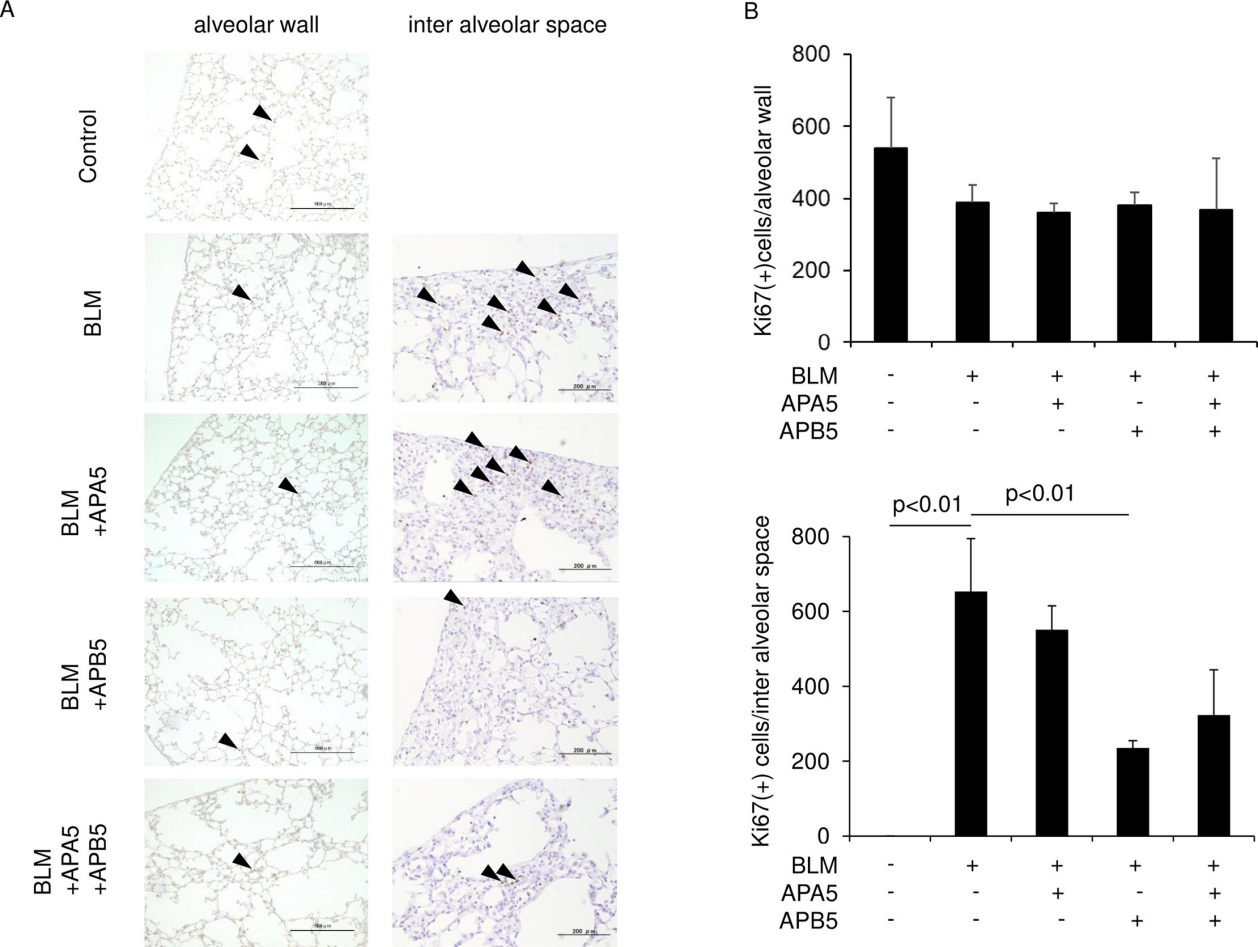
APB5, but not APA5, decreases proliferating cell numbers in inter-alveolar spaces of lungs. Mice were sacrificed on Day 14 after the administration of BLM. The lungs were examined by immunohistochemistry. (A) Ki-67 staining is shown. The administration of APB5 resulted in a lower number of Ki-67-positive cells in the inter-alveolar spaces than in BLM-treated mice, whereas that of APA5 did not. In the alveolar wall, there was no significant difference in the number of Ki-67-positive cells. Magnification ×100 for alveolar wall and ×200 for inter-alveolar space. (B) Ki-67-positive nuclei in the inter-alveolar spaces and in the alveolar wall of whole sections were counted. The treatment with APB5 reduced the number of Ki-67-positive cells in the inter-alveolar spaces significantly more than control IgG. Data are presented as means ± SEM. N = 3 for each group.

We investigated the mechanisms responsible for the stronger anti-fibrotic effects of APB5 over those of APA5. The proliferation of mesenchymal cells mainly in fibrotic areas was analyzed by immunohistochemistry for Ki-67. We previously confirmed in similar experiments that most proliferating mesenchymal cells were fibroblasts [19]. As shown in Fig 5A and 5B, the administration of APB5, but not APA5, significantly decreased the number of Ki-67-positive proliferating cells in the inter-alveolar spaces, not alveolar wall, of lungs treated with BLM. There were no additional effects of APA5 on the reductions observed in the number of Ki-67-positive cells by APB5.

### Effects of APA5 and APB5 on inflammation in lungs treated with BLM

To clarify the effects of APA5 and APB5 on inflammation in lungs treated with BLM, BAL fluid was collected on Days 7 and 14 and analyzed. On Day 7, the numbers of all cells and neutrophils were higher in BLM-treated mice than in control mice. APA5 increased the number of neutrophils, whereas APB5 decreased it. On Day 14, the numbers of all cells, macrophages, lymphocytes, and neutrophils were higher in BLM-treated mice than in control mice. Similarly, APB5 decreased the number of neutrophils. APB5 also slightly reduced the number of lymphocytes (Table 1).

**Table 1:** Effects of APA5 and APB5 on the classification of BAL cells in mice treated with bleomycin.

Days after BLM treatment	All cells (×106)	Cell classification (%)		
		Macrophages	Lymphocytes	Neutrophil
Day 7				
NS	0.53±0.12	91.9±9.46	3.20±0.02	2.26±0.03
BLM+IgG	1.02±0.07	75.63±5.14	4.59±0.07	10.06±0.24
BLM+APA5	1.24±0.28	67.16±10.7	7.72±0.3	21.72±1.52 ^⋆^
BLM+APB5	0.94±0.13	72.96±8.68	13.13±0.49	3.39±0.04 **
Day 14				
NS	0.43±0.15	88.0±8.69	2.12±0.01	0±0
BLM+IgG	2.12±0.4	76.6±23.8	12.6±1.04	5.27±0.17
BLM+APA5	2.44±0.14	80.7±7.81	12.7±0.81	1.94±0.02
BLM+APB5	1.93±0.28	83.3±20.7	8.36±0.35	1.41±0.03 *

Mice were treated with bleomycin (BLM) as described in the Materials and Methods section. APA5, APB5, and IgG as the control were intraperitoneally injected every other day. Mice were sacrificed on Days 7 and 14, and BAL fluid was collected. All and differential cell counting were performed. On Day 7, APA5 significantly increased the number of neutrophils and APB5 decreased it. On Day 14, APB5 decreased the number of neutrophils. Data are presented as means ± SEM. n = 4 for the control and n = 5–7 for the BLM treatment group.

### Evaluation of epithelial injury in lungs treated with anti-PDGFR antibodies in the BLM-induced pulmonary fibrosis model

**Fig 6:**
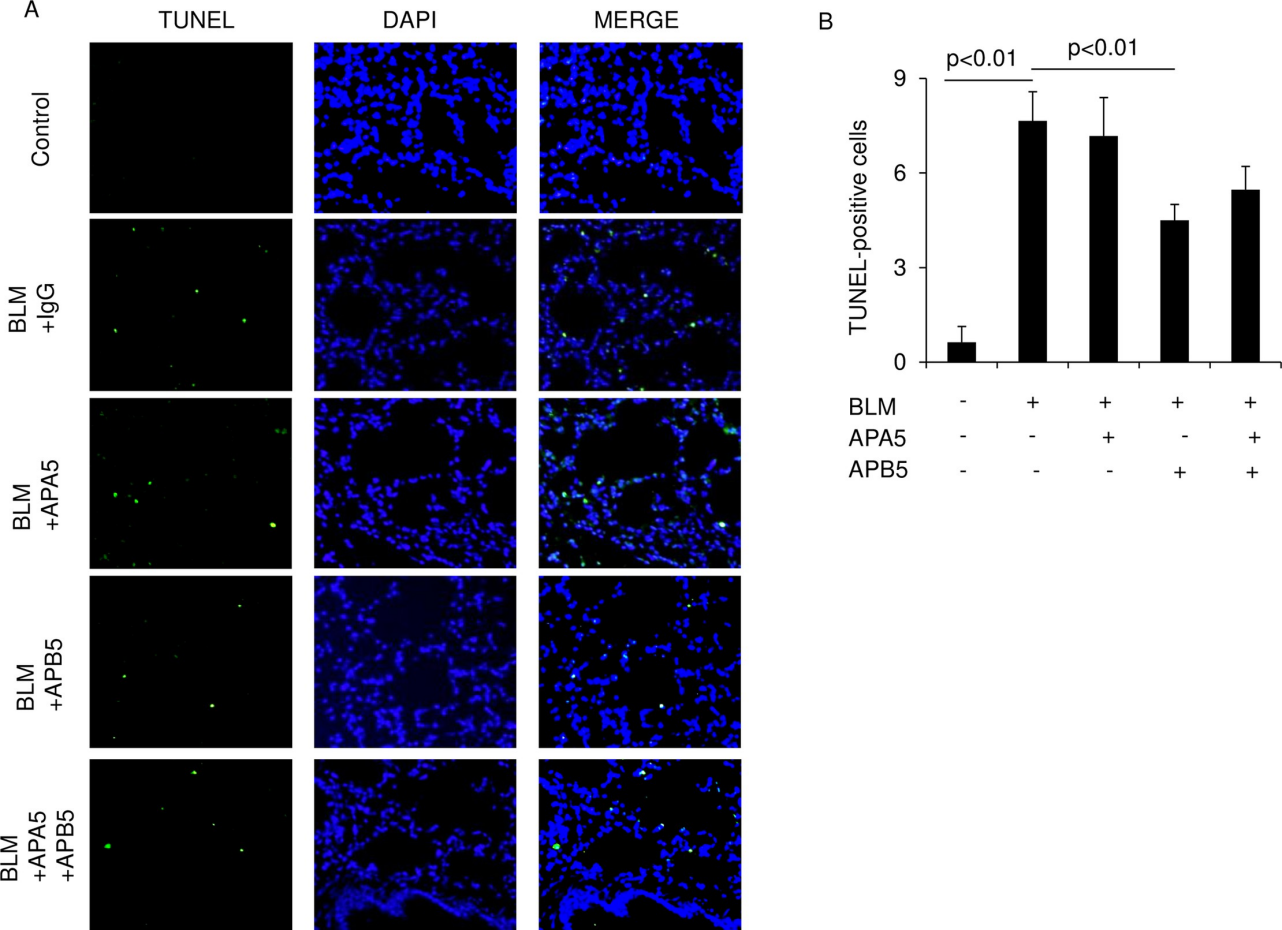
APB5, but not APA5, decreases apoptosis in alveolar epithelial cells in lungs of BLM-treated mice. Regarding the analysis of injured epithelial cell death after the BLM treatment on Day 14, sections were stained using the terminal deoxynucleotidyl transferase-mediated dUTP-biotin nick end labeling (TUNEL) method. (A) Representative pictures of TUNEL staining are shown. Nuclei were counterstained with 4’-6-diamidino-2-phenylindole (DAPI). Magnification ×400. (B) TUNEL-positive cells/field were counted from 6 random fields per section at ×200. BLM-treated mice showed significant increases in the number of TUNEL-positive cells in the lungs of mice on Day 14. The administration of APA5 did not change the number of TUNEL-positive cells from that in BLM-treated mice, whereas that of APB5 significantly decreased them. Data are presented as means ± SEM. N = 2 for the control and 3 for the BLM treatment groups.

**Fig 7:**
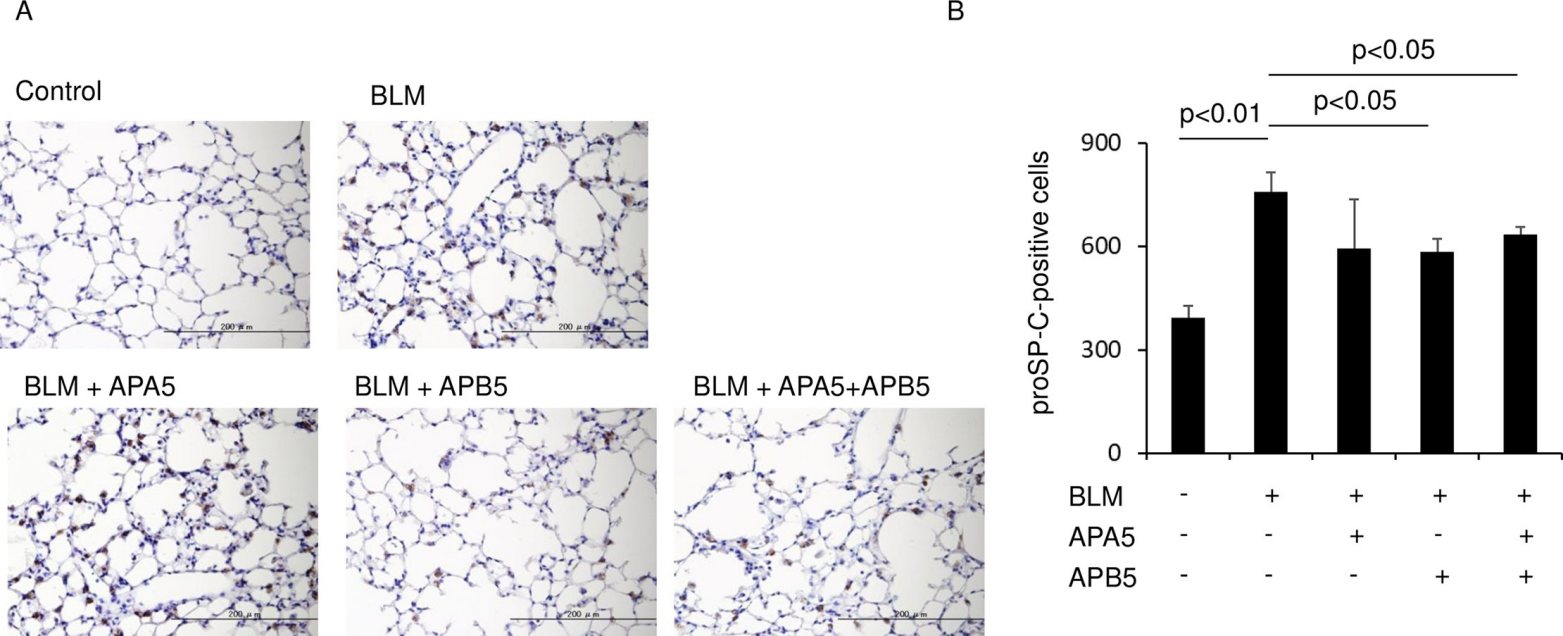
Administration of APB5, but not APA5, decreases the hyperplasia of alveolar type 2 epithelial cells induced by BLM. In the analysis of alveolar type 2 epithelial cells after the BLM treatment, sections were stained with an anti-pro SP-C antibody on Day 14. (A) Representative pictures are shown. The BLM treatment induced hyperplasia and increased the number of SP-C-positive type 2 epithelial cells. The administration of APB5 markedly decreased the hyperplasia of type 2 epithelial cells induced by BLM. Magnification ×400. (B) ProSP-C-positive nuclei in 3 random fields were counted at ×100. The BLM treatment increased pro SP-C-positive alveolar type 2 epithelial cells in the lungs on Day 14. The administration of APA5 did not inhibit the increase in cell counts, while that of APB5 significantly inhibited it. Data are presented as means ± SEM. N = 3 for each group.

To examine whether APA5 or APB5 affects alveolar epithelial cell injury induced by BLM, we used the TUNEL assay to assess apoptosis in alveolar epithelial cells on Day 14. BLM-treated mice showed a significant increase in TUNEL-positive cells in the lungs. The administration of APA5 did not change the number of apoptotic cells from those in BLM-treated mice, whereas that of APB5 significantly decreased their number (Fig 6A and 6B). We then assessed alveolar type 2 epithelial cells in the lungs by immunohistochemical staining for proSP-C. The BLM treatment caused hypertrophy and increases in the number of proSP-C-positive alveolar type 2 epithelial cells on Day 14, presumably indicating epithelial injury and regeneration. The administration of APA5 did not change these numbers from those in BLM-treated mice. However, the administration of APB5 significantly inhibited hypertrophy and increases in the number of alveolar type 2 epithelial cells (Fig 7A and 7B).

### Expression of PDGFR-α and -β in lungs treated with BLM

**Fig 8:**
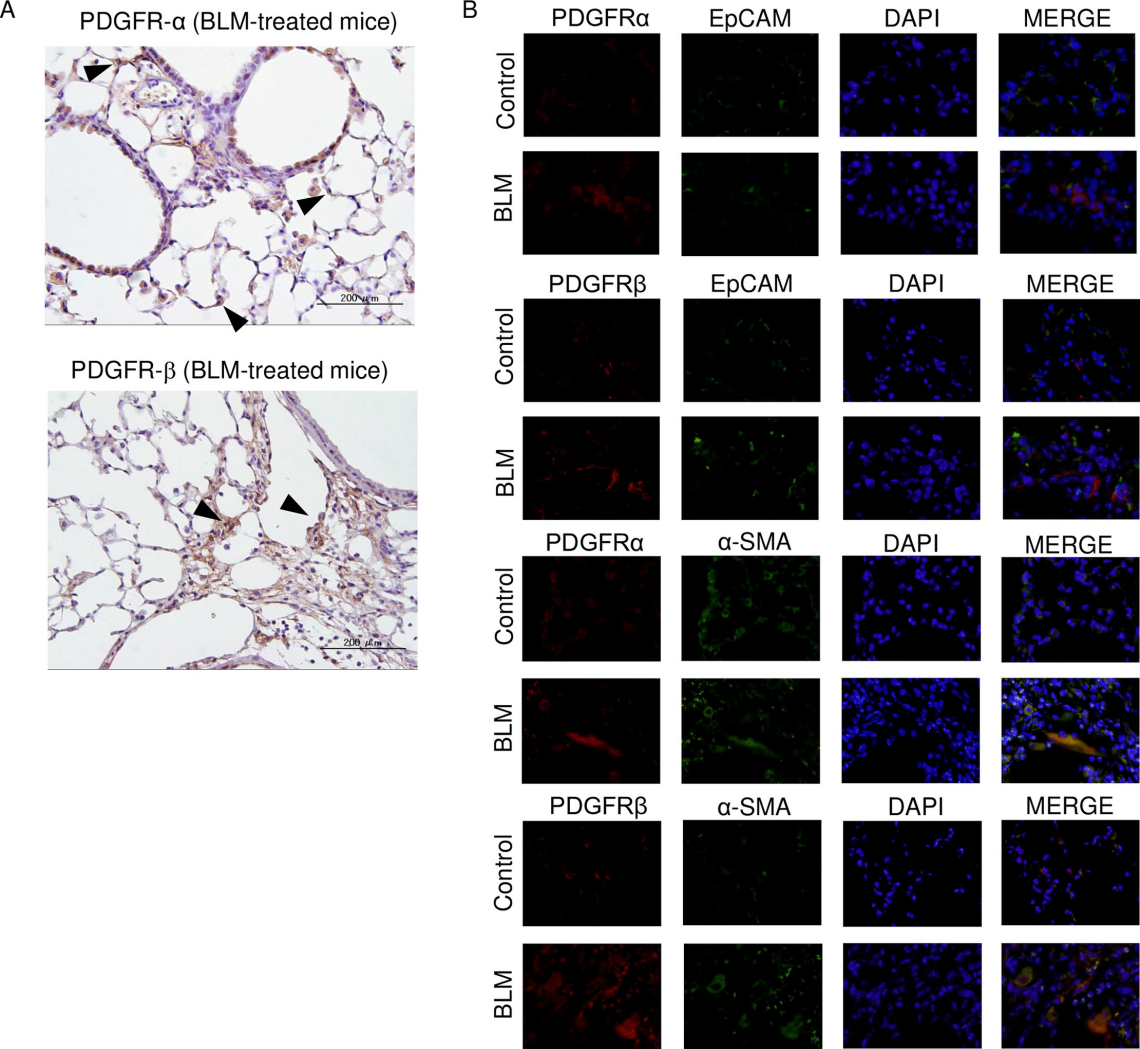
Differential expression of PDGFR-α and -β in fibrotic lungs. (A)The expression of PDGFRs in the lungs of BLM-treated mice was examined. Staining for PDGFRs was detected in fibrotic lungs. Several cells on alveolar wall showed weak staining for PDGFR-α. PDGFR-β was not clearly detected on alveolar wall. Mesenchymal cells clustering in fibrotic regions showed strong staining for PDGFR-α and -β; however, PDGFR-β was more strongly stained than PDGFR-α. (B) The double-staining with anti-PDGFR antibody and anti-EpCAM or anti-α SMA antibody. PDGFR-α and -β were expressed on α-SMA-positive fibroblasts, not EpCAM-positive lung epithelial cells.

To clarify the different effects of APA5 and APB5 *in vivo*, we investigated the expression patterns of PDGFRs in the lungs of BLM-treated mice. The staining of PDGFR-α was weakly detected in the alveolar wall (Fig 8A). On the other hand, fibrotic areas in lungs treated with BLM showed markedly stronger staining for PDGFR-β than for PDGFR-α possibly in mesenchymal cells as well as alveolar macrophages. We then examined whether PDGFR-α and -β expressed on lung fibroblasts or epithelial cells by immunohistochemistry. We performed the double-staining with anti-PDGFR antibody and anti-EpCAM or anti-α SMA antibody. As shown in Fig 8B, PDGFR-α and -β were expressed on α-SMA-positive fibroblasts, not EpCAM-positive lung epithelial cells.

### Late treatment with APB5 attenuates pulmonary fibrosis in BLM-treated mice

**Fig 9:**
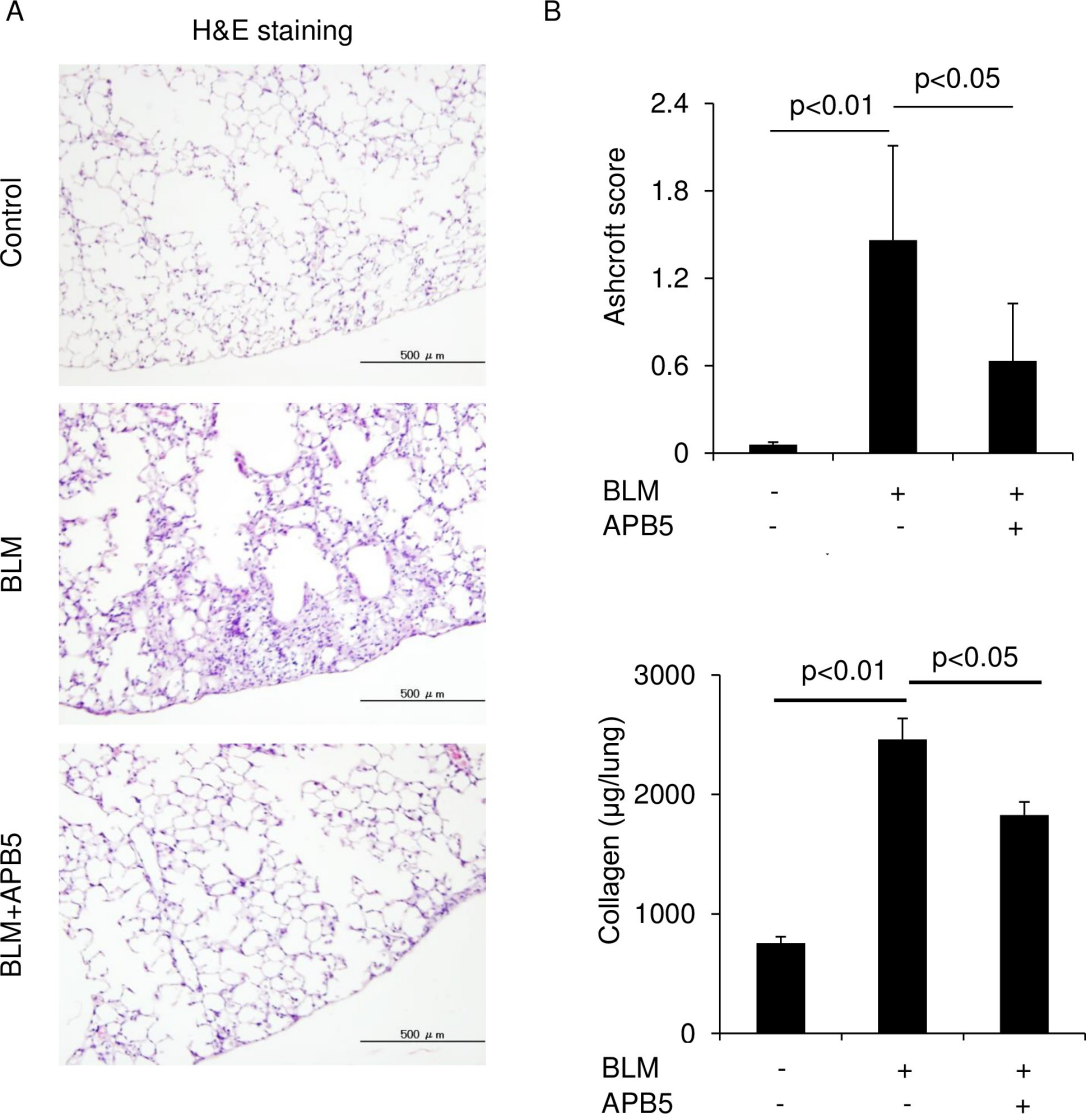
Late treatment with APB5 attenuates pulmonary fibrosis in BLM-treated mice. Mice were treated with BLM as described in the Materials and Methods section. APB5 and IgG as the control were intraperitoneally injected every other day from Day 15 to 27 (late treatment). Mice were sacrificed on Day 28. (A) Representative pictures are shown. The late treatment with APB5 attenuated pulmonary fibrosis in BLM-treated mice. (B) APB5 significantly decreased the Ashcroft score and the collagen content from that in BLM-treated mice. Data are presented as means ± SEM. N = 4 for the control and N = 4–7 for the BLM treatment groups.

To examine the therapeutic anti-fibrotic activity of APB5, we injected 1 mg of APB5 every other day from Days 15 to 27(late treatment schedule) into the BLM model. As shown in Fig 9, APB5 significantly decreased pulmonary fibrosis and the collagen content of the lungs.

## Discussion

In the present study, we examined the effects of the anti-PDGFR-α Ab APA5 and anti-PDGFR-β Ab APB5 on the biological activities of lung fibroblasts *in vitro* and on BLM-induced pulmonary fibrosis *in vivo*. Although the inhibitory effects of APA5 and APB5 on the proliferation of lung fibroblasts were similar, APB5 showed a significantly higher potential than APA5 to inhibit pulmonary fibrosis *in vivo*. Our results clearly indicate that the blocking approach to PDGFR-β is superior to that to PDGFR-α for treating pulmonary fibrosis. Furthermore, specific targeting to PDGFR-β may be one of the promising therapeutic approaches for patients with pulmonary fibrosis.

Fibroblasts are involved in damaged tissue repair and fibrogenesis through PDGFR signaling. They are heterogenous, expressing either PDGFR-α, -β, or both [20]. PDGFs binding to PDGFR-α and -β promote receptor dimerization, which activate PDGFR signaling. PDGF-AA has been reported to interact with only PDGFR-αα. On the other hand, PDGF-BB is considered to interact with PDGFR-αα, PDGFR-ββ, and PDGFR-αβ. However, a few interactions have been demonstrated *in vivo*, namely, those of PDGF-AA with PDGFR-αα and PDGF-BB with PDGFR-ββ [21]. Regarding the roles of PDGFRs in pulmonary fibrosis, there are a few papers published. Zhou Y et al. reported the expressions of PDGFRs in murine BLM-model [22]. They described that the level of PDGFR-α mRNA was not changed among days 4, 8 and 16 after a BLM administration. However, PDGFR-β mRNA was reported to be once decreased on day 8 and recovered on day14. On the other hand, Yoshida M et al. reported that in vivo gene transfer of an extracellular domain of PDGFR-β ameliorated BLM-induced pulmonary fibrosis in mice [23]. These data suggest that the roles of PDGFRs in pulmonary fibrosis are still unclear. To our best knowledge, the present study is a first report with blocking antibodies to analyze describing the roles of PDGFRs in pulmonary fibrosis in a mouse model.

First, we confirmed the specificity of APA5 and APB5 for PDGFRs by an immunoblotting. The data clearly showed that APA5 inhibited the signaling pathway of PDGFR-α, not PDGFR-β. However, APB5 affected the signaling of PDGFR-α in addition to PDGFR-β, although the effects were weaker. The binding specificity of APA5 and APB5 for PDGFR-α and -β is demonstrated in the previous papers [24, 16]. Because APA5, but not APB5, almost completely inhibited the proliferation of fibroblasts induced by PDGF-AA, and APB5, but not APA5, inhibited the growth induced by PDGF-BB, APA5 biologically inhibits the signaling mainly through PDGFR-α and APB5 biologically inhibits the signaling mainly through PDGFR-β.

On the other hand, PDGFR-α expressed on fibroblasts are reported to be important for normal tissue repair against fibrogenesis. Prasad et al. showed that PDGFR-α in fibroblasts from normal human lungs played essential roles in epithelial repair, and fibroblasts from the lungs of IPF patients expressed low PDGFR-α levels and exhibited deficient repair responses [25]. Chen L et al. found the dynamic regulation of PDGFR-α expression in fibroblasts during realveolization in the adult lungs of mice [26]. Fibroblasts expressing PDGFR-α have been shown to localize to the alveolar entry ring [27]. In the normal murine lung, genetic fate mapping with the forkhead transcription factor Foxd1 transiently expressed in the mesenchymal progenitor lineage in embryogenesis showed that resident fibroblasts (collagen-positive cells) expressed PDGFR-α, but not PDGFR-β [20]. They were activated when epithelial cells were denuded, and promoted the re-epithelization or accumulation of fibroblasts [28, 29]. In the present study, the administration of APA5 promoted the infiltration of inflammatory cells, particularly neutrophils, in BALF, but did not conclusively affect pulmonary fibrosis. We also showed that the patterns of PDGFR-α and -β expression were histologically different in the lungs of BLM-treated mice. Since murine alveolar type 2 epithelial cells in primary cultures were confirmed not to express PDGFR-α or -β, PDGFR-α-positive fibroblasts may exist adjacent to the alveolar spaces of the lungs, maintain the lung structure, and serve in epithelial repair through PDGFR-α signaling. Although the role of PDGFR-α in pulmonary fibrosis is still unclear, APA5 may inhibit the protective function of PDGFR-α-positive resident fibroblasts in epithelial injury and enhance inflammatory responses by blocking PDGFR-α on fibroblasts.

On the other hand, PDGFR-β in fibroblasts has been suggested to play a critical role in pulmonary fibrosis. Previous studies demonstrated that the stimulation of PDGFR-β in fibroblasts was essential for the proliferation and migration of fibroblasts in lung fibrogenesis. We previously reported that PDGF-BB induced the proliferation of fibroblasts more strongly than PDGF-AA [6]. In our study, the administration of APB5 markedly decreased the number of Ki-67-positive mesenchymal cells in fibrotic lesions. Hung et al. showed that 68% of lung myofibroblasts in BLM-treated mice were derived from pericytes that only expressed PDGFR-β, indicating an elevation in the relative expression of PDGFR-β in fibrotic lungs. Pericytes were denuded from vascular endothelial cells after the BLM treatment, which resulted in the extravasation of inflammatory cells [20]. In the present study, APB5 also decreased the infiltration of inflammatory cells, which may be related to the inhibitory effects of the activation of fibroblasts through the blockade of PDGFR-β.

Apart from fibroblasts, fibrocytes have been proposed as other candidates that contribute to pulmonary fibrosis through PDGFR. The migration of bone marrow-derived fibrocytes into damaged lungs has been reported to contribute to fibrosis. We showed that the expression of PDGFR-β was significantly stronger in fibrocytes from IPF patients than in those from healthy volunteers, and the migration of fibrocytes in the lungs of BLM-treated mice was inhibited more strongly by APB5 than by APA5 [30]. APB5 may ameliorate fibrosis in the lungs of BLM-treated mice, at least in part, by inhibiting the migration of fibrocytes.

Guzy et al. recently reported that FGF2 knockout mice showed increased mortality in response to BLM [31]. FGF receptors expressed in mouse and human alveolar epithelial cells is required for epithelial recovery. We also found that the blockade of pan-FGFRs with a specific inhibitor resulted in greater injury to alveolar epithelial cells and the mortality of mice treated with BLM (manuscript in preparation). In the present study, alveolar type 2 epithelial cells did not express PDGFR-α or -β, and the treatment with APA5 or APB5 did not significantly affect the mortality of mice. These results suggest that in contrast to FGFR, the blockade of PDGFRs is not harmful for alveolar epithelial cells.

Furthermore, in our experiments, the administration of APB5 during the late fibrotic phase also effectively ameliorated pulmonary fibrosis. In the model of BLM-induced pulmonary fibrosis, the early phase (Days 1–14) represents inflammation and the impaired repair of epithelial cell injury, while the late phase (Days 15–28) represents the accumulation of fibroblasts and collagen deposition. The effectiveness of the late administration of APB5 implies that APB5 did not only improve acute injury, but also inhibited chronic fibrosis, indicating the therapeutic anti-fibrotic effects of the anti-PDGFR-β antibody.

In summary, PDGFR-α and -β exerted different effects on BLM-induced pulmonary fibrosis in mice. In fibrotic lungs, PDGFR-β-dependent pathways appear to more strongly contribute to the progression of pulmonary fibrosis than those of PDGFR-α, and the specific inhibition of PDGFR-β, but not PDGFR-α effectively inhibits pulmonary fibrosis. Therefore, the anti-PDGFR-β antibody may be a useful therapeutic modality without lung toxicity for patients with IPF.

## Supporting information

S1 Fig FACS analysis of PDGFR-α and -β of lung fibroblasts and epithelial cells of C57B/L6 mouse. The expression rate of PDGFR-α and -03B2 were shown. The analysis of CCL206 lung fibroblasts were also performed and similar results were obtained.
(TIF)

S2 Fig Original uncropped data for Western blotting of Fig 2. (TIF)

## References

[1] Richeldi L, Collard HR, Jones MG. Idiopathic pulmonary fibrosis. Lancet. 2017; 389: 1941–1952. 10.1016/S0140-6736(17)30866-8 28365056

[2] Nishioka Y. Pathogenesis of IPF. Is abnormal repair of epithelial damage involved in the basic pathogenesis of this disease? In: Nakamura H, Aoshiba K, editors. Idiopathic Pulmonary Fibrosis, Springer; 2016 pp43–58.

[3] Nishioka Y, Azuma M, Kishi M, Aono Y. Targeting platelet-derived growth factor as a therapeutic approach in pulmonary fibrosis. J Med Invest. 2013; 60:175–183. 24190033 10.2152/jmi.60.175

[4] Bonner JC. Regulation of PDGF and its receptors in fibrotic diseases. Cytokine Growth Factor Rev. 2004; 15: 255–273. 10.1016/j.cytogfr.2004.03.006 15207816

[5] Abdollahi A, Li M, Ping G, Plathow C, Domhan S, Kiessling F, et al Inhibition of platelet-derived growth factor signaling attenuates pulmonary fibrosis. J Exp Med. 2005; 201: 925–935. 10.1084/jem.20041393 15781583 PMC2213091

[6] Aono Y, Nishioka Y, Inayama M, Ugai M, Kishi J, Uehara H, et al Imatinib as a novel antifibrotic agent in bleomycin-induced pulmonary fibrosis in mice. Am J Respir Crit Care Med. 2005; 171: 1279–1285. 10.1164/rccm.200404-531OC 15735062

[7] Daniels CE, Wilkes MC, Edens M, Kottom YJ, Murphy SJ, Limper AH, et al Imatinib mesylate inhibits the profibrogenic activity of TGF-β and prevents bleomycin-mediated lung fibrosis. J Clin Invest. 2004; 114: 1308–1316 10.1172/JCI19603 15520863 PMC524221

[8] Daniels CE, Lasky LA, Limper AH, Mieras K, Gabor E, Schroeder DR, et al Imatinib treatment for idiopathic pulmonary fibrosis. randomized placebo-controlled trial results Am J Respir Crit Care Med. 2010; 181: 604–610. 10.1164/rccm.200906-0964OC 20007927

[9] Richeldi L, du Bois RM, Raghu G, Azuma A, Brown KK, Costabel U, et al INPULSIS Trial Investigators. Efficacy and safety of Nintedanib in idiopathic pulmonary fibrosis. N Engl J Med. 2014; 370: 2071–2082. 10.1056/NEJMoa1402584 24836310

[10] Wollin L, Neugebauer J, Ostermann A, Holweg A, Wex E, Ehinger K, et al Sustained inactivation of human lung fibroblasts by Nintedanib [abstract]. Am J Respir Crit Care Med. 2013; 187: A3378.

[11] Betsholtz C. Insight into the physiological functions of PDGF through genetic studies in mice. Cytokine Growth Factor Rev. 2004; 15: 215–228. 10.1016/j.cytogfr.2004.03.005 15207813

[12] Boström H, Willetts K, Pekny M, Levéen P, Lindahl P, Hedstrand H, et al PDGF-A signaling is a critical event in lung alveolar myofibroblast development and alveogenesis. Cell. 1996; 85: 863–873. 8681381 10.1016/s0092-8674(00)81270-2

[13] Phan SH, Varani J, Smith D, Rat lung fibroblast collagen metabolism in bleomycin-induced pulmonary fibrosis. J Clin Invest 1985; 76: 241–247 10.1172/JCI111953 2410457 PMC423755

[14] Corti M, Brody AR, Harrison JH. Isolation and primary culture of murine alveolar type II cells. Am J Respir Cell Mol Biol. 1996;14(4):309–315. 10.1165/ajrcmb.14.4.8600933 8600933

[15] Harrison JH, Lazo JS. High dose continuous infusion of bleomycin in mice: a new model for drug-induced pulmonary fibrosis. J Pharmacol Exp Ther. 1987; 243: 1185–1194. 2447265

[16] Sano H, Sudo T, Yokode M, Murayama T, Kataoka H, Tanakura N, et al Functional blockade of platelet-derived growth factor receptor-β but not of receptor-α prevents vascular smooth muscle cell accumulation in fibrous cap lesions in apolipoprotein E-deficient mice. Circulation. 2001; 103: 2955–2960. 11413086 10.1161/01.cir.103.24.2955

[17] Sano H, Ueda Y, Takakura N, Takemura G, Doi T, Kataoka H, et al Blockade of platelet-derived growth factor receptor-β pathway induces apoptosis of vascular endothelial cells and disrupts glomerular capillary formation in neonatal mice. Am J of Pathol. 2002; 161: 135–143. 12107098 10.1016/s0002-9440(10)64165-x PMC1850709

[18] Ashcroft T, Simpson JM, Timbrell V. Simple method of estimating severity of pulmonary fibrosis on a numerical scale. J Clin Pathol. 1988; 41: 467–470. 3366935 10.1136/jcp.41.4.467 PMC1141479

[19] Azuma M, Nishioka Y, Aono Y, Inayama M, Makino H, Kishi J, et al Role of alpha1-acid glycoprotein in therapeutic antifibrotic effects of imatinib with macrolides in mice. Am J Respir Crit Care Med. 2007; 176: 1243–1250. 10.1164/rccm.200702-178OC 17717205

[20] Hung C, Linn G, Chow YH, Kobayashi A, Mittelsteadt K, Altemeier WA, et al Role of lung pericytes and resident fibroblasts in the pathogenesis of pulmonary fibrosis. Am J Respir Crit Care Med. 2013; 188: 820–830. 10.1164/rccm.201212-2297OC 23924232 PMC3826269

[21] Andrae J, Gallini R, Betsholtz C. Role of platelet-derived growth factors in physiology and medicine. Genes Dev. 2008; 22: 1276–1312. 10.1101/gad.1653708 18483217 PMC2732412

[22] Zhuo Y1, Zhang J, Laboy M, Lasky JA. Modulation of PDGF-C and PDGF-D expression during bleomycin-induced lung fibrosis. Am J Physiol-Lung Cell Mol Physiol. 2004; 286: 182–188. 10.1152/ajplung.00083.2003 12972405

[23] Yoshida M1, Sakuma-Mochizuki J, Abe K, Arai T, Mori M, Goya S, et al In vivo gene transfer of an extracellular domain of platelet-derived growth factor beta receptor by the HVJ-liposome method ameliorates bleomycin-induced pulmonary fibrosis. Biochem Biophys Res Commun. 1999; 265(2): 503–508. 10.1006/bbrc.1999.1647 10558898

[24] Takakura N, Yoshida H, Kunisada T, Nishikawa S, Nishikawa SI. Involvement of platelet-derived growth factor receptor-alpha in hair canal formation. J Invest Dermatol. 1996; 107(5): 770–7. 8875964 10.1111/1523-1747.ep12371802

[25] Prasad S, Hogaboam CM, Jarai G. Deficient repair response of IPF fibroblasts in a co-culture model of epithelial injury and repair. Fibrogenesis Tissue Repair. 2014; 7: 7 10.1186/1755-1536-7-7 24834127 PMC4021590

[26] Chen L, Acciani T, Le Cras T, Lutzko C, Perl AK. Dynamic regulation of platelet-derived growth factor receptor α expression in alveolar fibroblasts during realveolarization. Am J Respir Cell Mol Biol. 2012; 47: 517–527. 10.1165/rcmb.2012-0030OC 22652199 PMC3488620

[27] McGowan SE, Grossmann RE, Kimani PW, Holmes AJ. Platelet-derived growth factor receptor-alpha-expressing cells localize to the alveolar entry ring and have characteristics of myofibroblasts during pulmonary alveolar septal formation. Anat Rec (Hoboken). 2008; 291: 1649–1661. 18833569 10.1002/ar.20764

[28] Barkauskas CE, Cronce MJ, Rackley CR, Bowie EJ, Keene DR, Stripp BR, et al Type 2 alveolar cells are stem cells in adult lung. J Clin Invest. 2013; 123: 3025–3036. 10.1172/JCI68782 23921127 PMC3696553

[29] Tsukui T, Ueha S, Abe J, Hashimoto S, Shichino S, Shimaoka T, et al Qualitative rather than quantitative changes are hallmarks of fibroblasts in bleomycin-induced pulmonary fibrosis. Am J Pathol. 2013; 183: 758–773. 10.1016/j.ajpath.2013.06.005 23886891

[30] Aono Y, Kishi M, Yokota Y, Azuma M, Kinoshita K, Takezaki A, et al Role of PDGF/PDGFR Axis in the Trafficking of Circulating Fibrocytes in Pulmonary Fibrosis. Am J Respir Cell Mol Biol. 2014; 51: 793–801. 10.1165/rcmb.2013-0455OC 24885373

[31] Guzy RD1, Stoilov I, Elton TJ, Mecham RP, Ornitz DM. Fibroblast growth factor 2 is required for epithelial recovery, but not for pulmonary fibrosis, in response to bleomycin. Am J Respir Cell Mol Biol. 2015; 52: 116–128. 10.1165/rcmb.2014-0184OC 24988442 PMC4370255

