# Supplementary figures and images for: Blockade of platelet-derived growth factor receptor-β, not receptor-α ameliorates bleomycin-induced pulmonary fibrosis in mice
Source: PLoS One. 2018 Dec 31;13(12):e0209786. doi: 10.1371/journal.pone.0209786 (PMC6312310; doi:10.1371/journal.pone.0209786)

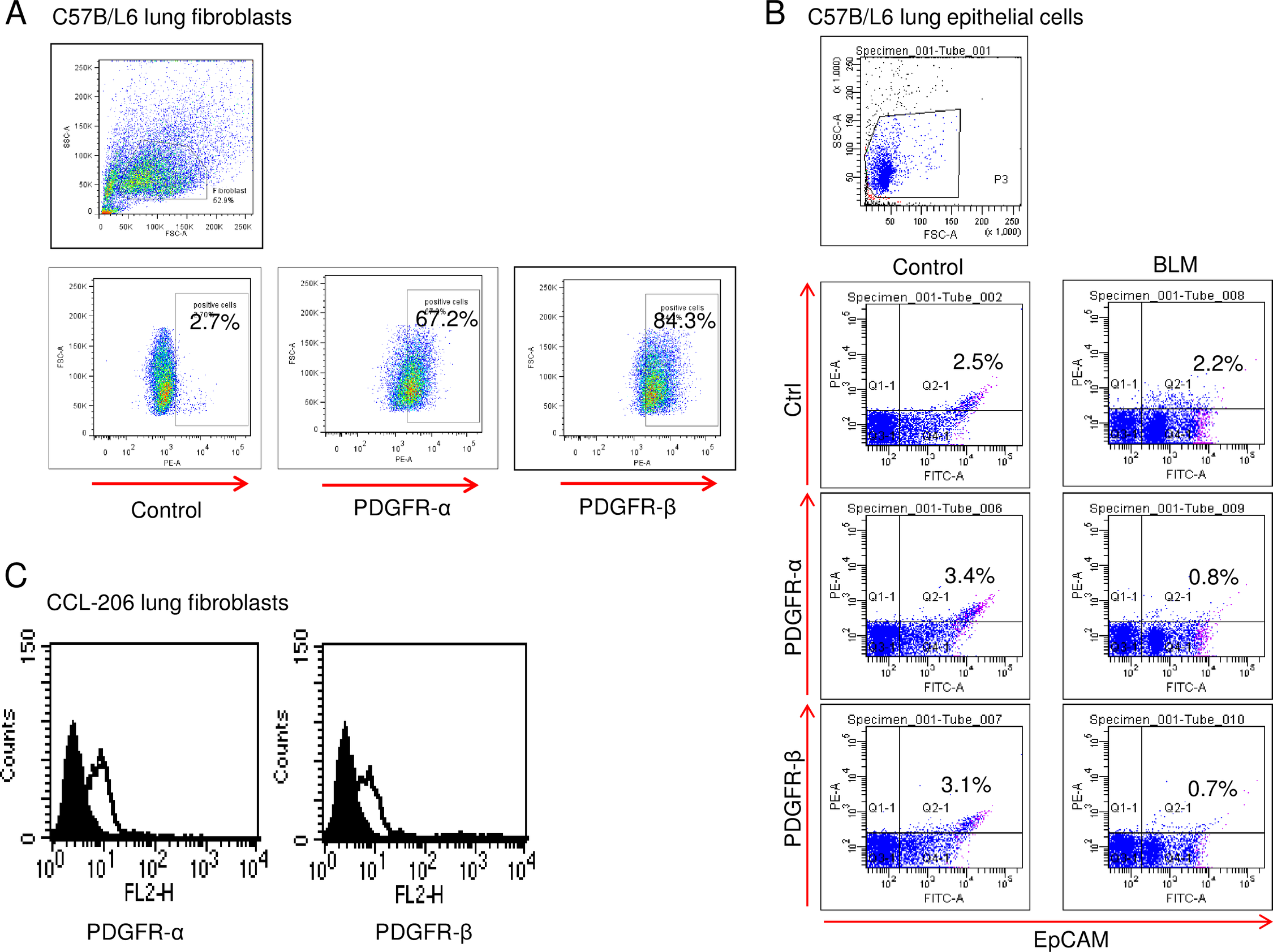

Supplement: S1 Fig — The expression rate of PDGFR-α and -03B2 were shown. The analysis of CCL206 lung fibroblasts were also performed and similar results were obtained. (TIF) [file pone.0209786.s001.tif]

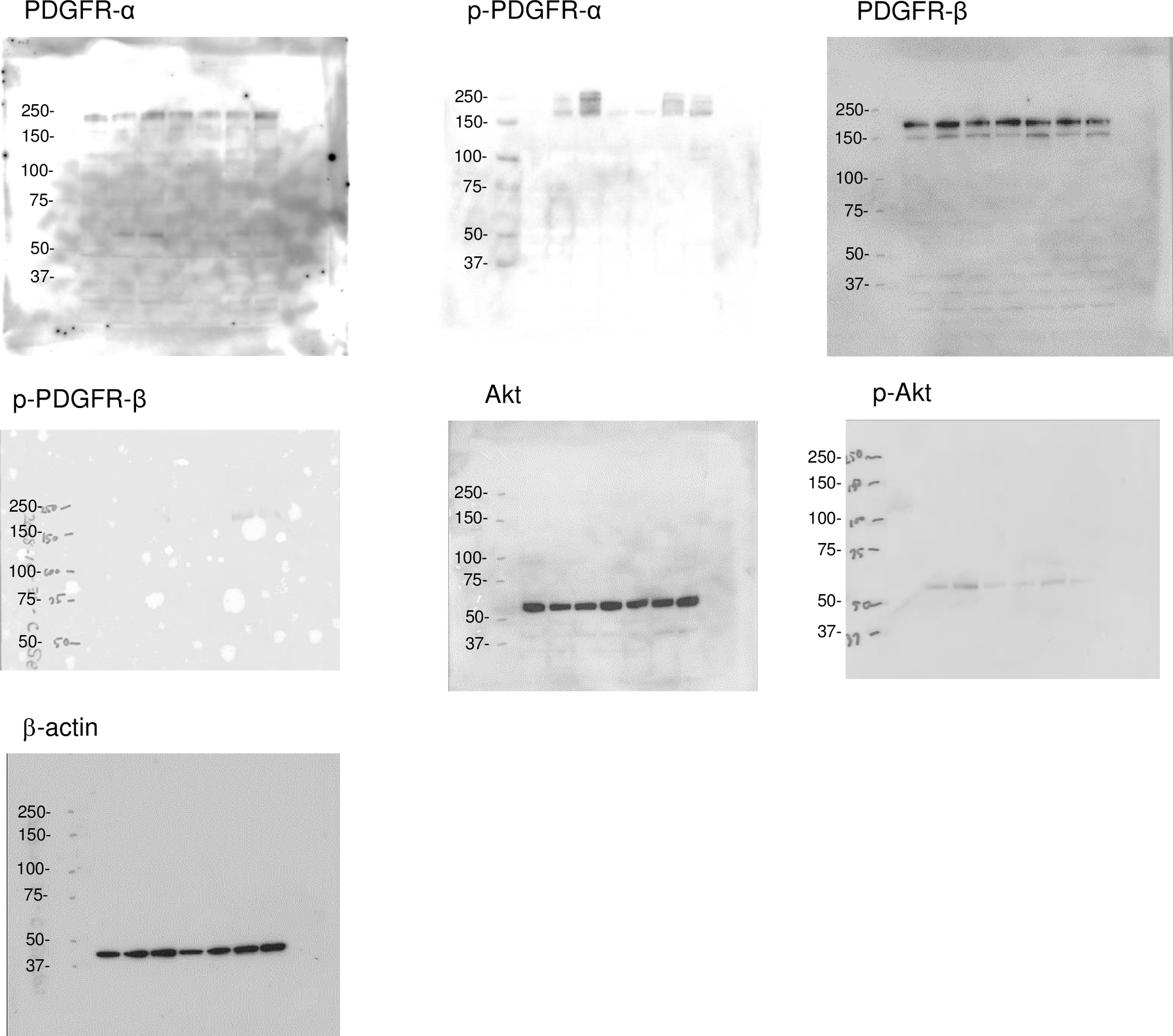

Supplement: S2 Fig — (TIF) [file pone.0209786.s002.tif]
